# METTL3-Mediated N6-Methyladenosine Modification Is Involved in the Dysregulation of NRIP1 Expression in Down Syndrome

**DOI:** 10.3389/fcell.2021.621374

**Published:** 2021-04-01

**Authors:** Weili Shi, Fan Yang, Ranran Dai, Yafei Sun, Yan Chu, Shixiu Liao, Bingtao Hao

**Affiliations:** ^1^Henan Provincial People’s Hospital, Medical Genetics Institute of Henan Province, Henan Provincial Key Laboratory of Genetic Diseases and Functional Genomics, People’s Hospital of Zhengzhou University, Zhengzhou, China; ^2^National Health Commission Key Laboratory of Birth Defect Prevention, Henan Key Laboratory of Population Defects Prevention, Zhengzhou, China; ^3^Zhengzhou Central Hospital, Department of Neurology, Zhengzhou, China; ^4^Guangdong Provincial Key Laboratory of Tumor Immunotherapy, Cancer Research Institute, School of Basic Medical Sciences, Southern Medical University, Guangzhou, China

**Keywords:** Down syndrome, NRIP1, m6A modification, METTL3, cerebral cortex

## Abstract

Down syndrome (DS) is a common genetic condition in which a person is born with an extra copy of chromosome 21. Intellectual disability is the most common characteristic of DS. N6-methyladenosine (m6A) is a common RNA modification that is implicated in many biological processes. It is highly enriched within the brain and plays an essential role in human brain development. However, the mRNA m6A modification in the fetal brain of DS has not been explored. Here, we report m6A mRNA profiles and mRNA expression profiles of fetal brain cortex tissue from DSs and controls. We observed that the m6A modification in DS brain tissues was reduced genome-wide, which may be due to decreased the m6A methyltransferase like 3 (METTL3) protein expression. The nuclear receptor-interacting protein 1 (*NRIP1/RIP140*) is coded by a highly conserved chromosome 21 (Hsa21) gene. Overexpression of NRIP1 is associated with mitochondrial dysfunction in DS. The NRIP1 mRNA increased in fetal brain tissues of DS, whereas the m6A modification of the NRIP1 mRNA significantly decreased. METTL3 knockdown reduced the m6A modification of NRIP1 mRNA and increased its expression, and an increase in NRIP1 m6A modification and a decrease in its expression were observed in METTL3-overexpressed cells. The Luciferase reporter assay confirmed that METTL3 regulates NRIP1 expression in an m6A-dependent manner. The decay rate of NRIP1 mRNA was significantly reduced in METTL3-knockdown cells but increased in METTL3-overexpressed cells. We proposed that the m6A modification of NRIP1 mRNA in DS fetal brain tissue is reduced, reducing its transcript degradation rate, resulting in abnormally increased expression of NRIP1, at least partially, in the DS brain. It provides a new mechanism for the molecular pathology of DS and leads to a new insight that may become therapeutically relevant.

## Introduction

N6-methyladenosine (m6A) is one of the most common and abundant internal modifications in polyadenylated mRNAs and long non-coding RNAs and plays critical roles in diverse biological processes (Cao et al., 2016; Li et al., 2017; Chen et al., 2019). The methylation of adenosine is catalyzed by an m6A methyltransferase complex containing METTL3 (methyltransferase like 3) and METTL14 (methyltransferase like 14). The demethylation is catalyzed by m6A demethylases FTO (ALKBH9) and ALKBH5. The methylation and demethylation dynamically control the RNA life cycle, affecting transcription, mRNA transport, splicing, stability, transcript abundance, and translation (Fu et al., 2014). It was reported that the m6A was highly enriched with the brain and markedly increased during human brain development (Meyer et al., 2012). The m6A profiling analysis of several fetal tissues, including the brain, revealed a dynamic m6A methylation landscape during human fetal development (Xiao et al., 2019). The m6A was predominantly enriched in transcripts related to neurogenesis and neuronal development (Chen et al., 2019). The characteristic of m6A in the brain suggested that it plays a critical role in brain development (Li et al., 2019).

Aberrant m6A modification has been linked to many diseases. ALKBH5-deficient male mice displayed an increased m6A in mRNA and impaired fertility resulting from increased apoptosis of spermatocytes (Zheng et al., 2013). The m6A modification has been reported related to major depressive disorder (Du et al., 2015), fragile X syndrome (Zhang et al., 2018), acute myeloid leukemia (Kwok et al., 2017), and Alzheimer disease (Han et al., 2020). Down syndrome (DS), or trisomy 21 (MIM: 190685), is the most common aneuploidy caused by triplication of human chromosome 21. One of DS’s common characteristics is an intellectual disability and early onset of Alzheimer disease (Wisniewski et al., 1985). The development of the brain in DS has been extensively studied (Dierssen, 2012; Lee et al., 2016; Shi et al., 2016; Arai et al., 2019), but the role of m6A in it is unclear.

Nuclear receptor-interacting protein 1 (NRIP1), also known as receptor-interacting protein 140 (RIP140), is encoded by the NRIP1 gene located on human chromosome 21. NRIP1 is a key regulator that modulates the transcriptional activity of many transcription factors, including the estrogen receptor. NRIP1 is expressed in the cortical and hippocampus areas of the brain, which is essential for maintaining cognitive functions (Nautiyal et al., 2013). Overexpression of NRIP1 is associated with DS, cancer, inflammation, and Alzheimer disease (Nautiyal et al., 2013; Izzo et al., 2014; Lapierre et al., 2015; Blondrath et al., 2016). In DS, NRIP1 is a key gene in regulating the mitochondrial pathway, and it is a good candidate for a potential therapeutic target (Izzo et al., 2014).

In this study, we profiled m6A modification of the human fetal cerebral cortex and identified the different m6A-modified transcripts between DS and control. We observed that the m6A modification in DS brain tissues was reduced in genome-wide, and the m6A methyltransferase METTL3 protein expression decreased in the samples. We have found a significant increase of NRIP1 transcript accompanied by a reduction in m6A modification levels in the fetal DS cortex tissues. Further experiments showed that the m6A methyltransferase METTL3 regulated NRIP1 expression in an m6A-dependent manner. The dysregulated NRIP1 was affected by METTL3 via regulating mRNA stability. This study provided a new mechanism to explain the abundance of NRIP1 in the DS brain, implicating the role of the RNA m6A modification in DS pathology.

## Materials and Methods

### Tissue Collection

All aborted fetuses were obtained after the donors signed a written informed consent. Three fetuses in the second trimester of pregnancy that were diagnosed as DS or two fetuses that were diagnosed as diploid via karyotype analysis were collected, respectively (18–25 gestational weeks). Cerebral cortex was dissected from these fetuses.

### m6A Methylated RNA Immunoprecipitation–Seq Assay and Data Analysis

N6-methyladenosine methylated RNA immunoprecipitation (MeRIP)–seq was carried out as described somewhere with modification (Dominissini et al., 2013). Specifically, total RNA was extracted with Trizol (Invitrogen). Polyadenylated RNA was isolated by Sigma GenElute mRNA Miniprep kit. Purified mRNA was randomly fragmented via RNA fragmentation Reagents (Millipore). m6A antibody (Synaptic Systems) was incubated with Magnetic Beads A/G Blend (Millipore). Afterward, fragmented mRNA was added to m6A antibody–beads mixture. The eluted RNA was extracted with acid phenol followed by standard ethanol precipitation. Then next-generation sequencing was carried out both on m6A IP and input samples. In brief, the TruSeq Stranded mRNA Sample Prep Kit (Illumina) was used for library preparation, and then the library was deeply sequenced on an Illumina HiSeq platform.

Raw data of each sample were trimmed by the Trimmomatic software to remove adaptor and low-quality bases. Then, the reads greater than 50 bp were aligned to the human genome hg19 reference. m6A peaks were called by MACS2 with the corresponding input sample worked as control. The motifs enriched in m6A peaks were analyzed by HOMER. The enrichment score was calculated via dividing the RPM in MeRIP-seq by input RNA-seq. m6A peaks were identified as differential m6A peaks as the fold change of m6A enrichment score was greater than 2. Distribution of m6A peaks were visualized by Integrative Genomics Viewer.

### RNA-Seq and Data Analysis

Total RNA was extracted with Trizol (Invitrogen). Poly(A) RNA purification was performed via GenElute mRNA Miniprep kit (Sigma). Library construction was conducted by TruSeq Stranded mRNA Sample Prep Kit (Illumina) and then sequenced on the Illumina HiSeq system.

Raw reads of each sample were trimmed to remove adaptor sequences and low-quality bases. The reads of RNA-seq were normalized using Cufflinks. Differentially expressed genes (DEGs) were identified by cuffdiff. DEGs were identified with fold change greater than 2 and adjusted *p*-value less than 0.05 as the thresholds. Gene Ontology (GO) analysis was performed via DAVID online.

### Cell Culture, Transfection, Plasmid, and siRNAs

HepG2 and HEK293T cell lines were purchased from Cell Bank of Chinese Academy of Sciences (Shanghai). HT22 was purchased from iCell Bioscience (Shanghai). HEK293T, HepG2, and HT22 cells were maintained in Dulbecco modified eagle medium supplemented with 10% fetal bovine serum and 1% PS (penicillin and streptomycin) under standard culture conditions (5% CO_2_, 37°C). Cell transfection was carried out essentially as described previously (Shi et al., 2016). pCMV3-METTL3, pCMV3-METTL14, pCMV3-YTHDF3, and pCMV3 control vectors were purchased from Sino Biological (Beijing). The catalytic mutant METTL3, pCMV3-METTL3-MUT, was generated via mutating the residues of 395–398 (D395A and W398A). The mutant primers were synthesized as follows: forward 5′-AGTTGTGATGGCTGCCCCACCCGCGGATATTCACATGGA ACTG-3′; reverse 5′-CAGTTCCATGTGAATATCCGCGGGTGG GGCAGCCATCACAACT-3′. The polymerase chain reaction (PCR) product was digested with *Dpn*I, and then transformation was further performed to generate the mutant METTL3. METTL3-siRNAs and negative control siRNA were purchased from RiboBio (Guangzhou). METTL3 siRNA 1 with target sequence: 5′-CTGCAAGTATGTTCACTATGA-3′; METTL3 siRNA 2 with target sequence: 5′-AGGAGCCAGCCAAGAAAT CAA-3′; METTL3 siRNA 3 with target sequence: 5′-GCACTTG GATCTACGGAAT-3′. Mettl3 siRNA 1 with target sequence: 5′-TCGGACACGTGGAGCTCTA-3′; Mettl3 siRNA 2 with target sequence: 5′-CTGGACGTCAGTATCTTGG-3′; Mettl3 siRNA 3 with target sequence: 5′-CCACTCAAGATGGGGTAGA-3′.

### Quantitative Real-Time PCR

Total RNA was extracted using Trizol (Invitrogen) and used for synthesis of cDNA by RevertAid First Strand cDNA Synthesis Kit (Thermo Scientific) according to manufacturer’s instruction. cDNA was synthesized and served as templates to perform quantitative PCR (qPCR) with SYBR Green Master Mix (Applied Biosystems) on Applied Biosystems Stepone Real-Time PCR System. The expression of the housekeeping gene GAPDH was used as an internal control. Primers are as follows: METTL3 forward: 5′-CAAGCTGCACTTCAGACGAA-3′; METTL3 reverse: 5′-GCTTGGCGTGTGGTCTTT-3′; GAPDH forward: 5′-GTCT CCTCTGACTTCAACAGCG-3′; GAPDH reverse: 5′-ACCACC CTGTTGCTGTAGCCAA-3′; NRIP1 forward: 5′-GGATCAGGT ACTGCCGTTGAC-3′; NRIP1 reverse: 5′-CTGGACCATTACTT TGACAGGTG-3′. Mettl3 forward: 5′-CTGGGCACTTGGA TTTAAGGAA-3′; Mettl3 reverse: 5′-TGAGAGGTGGTGTAG CAACTT-3′; Nrip1 forward: 5′-AGCAGGACAAGAGTCACAG AAAC-3′; Nrip1 reverse: 5′-TGTGATGATTGGCAGTATC TACG-3′; β-actin forward: 5′-GGCTGTATTCCCCTCCATCG-3′; β-actin reverse: 5′-CCAGTTGGTAACAATGCCATGT-3′.

### MeRIP–Quantitative PCR

The MeRIP-qPCR assay was conducted according to the instructions of standard protocol of EpiMark N6-Methyladenosine Enrichment Kit (NEB). Briefly, the RNA from cerebral cortex or cell lines was used for m6A immunoprecipitation with m6A antibody. The immunoprecipitated RNA was further reverse transcribed via RevertAid First Strand cDNA Synthesis Kit (Thermo Scientific) and then analyzed by qPCR analysis. The primers were the same as the primers used in quantitative real-time (RT)–PCR analysis.

### Western Blotting

Western blotting was performed as described previously (Shi et al., 2015). Specifically, proteins were separated by sodium dodecyl sulfate–polyacrylamide gel electrophoresis. Antibodies for detecting protein were diluted at 1:1,000 for anti-METTL3 antibody (Abcam), anti-FTO antibody (Abcam), an anti-NRIP1 antibody (Abcam), an anti-Mettl3 antibody (Abcam), an anti-Nrip1 antibody (Abcam), and 1:5,000 for anti-β-ACTIN antibody (Abcam). The horseradish peroxidase–labeled secondary antibody (Abcam) was diluted at 1:10,000 and detected with enhanced chemiluminescence NcmECL Ultra kit (NCM Biotech, China).

### RNA Stability Assays

HEK293T cells were transfected with pCMV3-METTL3 or METTL3 siRNAs in a 12-well plate. Twenty-four hours later, the cells were treated with actinomycin D at a final concentration of 5 μg/mL and were collected at different time points. The cells were collected, and total RNAs were extracted for reverse transcription, and levels were measured by quantitative RT-PCR. NRIP1 mRNA levels were plotted after normalization against GAPDH.

### Dual-Luciferase Reporter and Mutagenesis

Wild-type NRIP1 5′ UTR or mutant NRIP1 5′ UTR (m6A was replaced by T) was inserted into upstream of psiCHECK2 dual-luciferase vector. For dual-luciferase reporter assay, cells seeded in 24-well plates were cotransfected with wild-type or mutant NRIP1–5′ UTR and METTL3 or control, respectively. Forty-eight hours after transfection, the activities of firefly luciferase and Renilla luciferase in each well were tested via Dual-Luciferase Reporter Assay System (Promega) according to the manufacturer’s protocol.

### RNA m6A Quantification

Total RNA was extracted from cerebral cortex using Trizol (Invitrogen). The m6A RNA Methylation Quantification Kit (Epigentek) was used to detect the global m6A content in the total RNA according to manufacturer’s instruction. Two hundred nanograms of total RNA was used for each sample analysis. The absorbance was measured on a microplate reader at 450 nm. The percentage of m6A in total RNA can be calculated using the following formula: m6A% = [(sample OD – NC OD) ÷ S]/[(PC OD – NC OD) ÷ P] × 100%, where *S* is the amount of sample RNA in nanograms, and *P* is the amount of positive control in nanograms.

### Statistical Analysis

The data were expressed as mean ± SD. The statistical significance of differences was analyzed using Student *t*-test or two-way analysis of variance with GraphPad Prism 8 (GraphPad Software). *p* < 0.05 was considered statistically significant.

## Results

### RNA m6A Modification Globally Decreased in the Fetal Cerebral Cortex of DS

**FIGURE 1:**
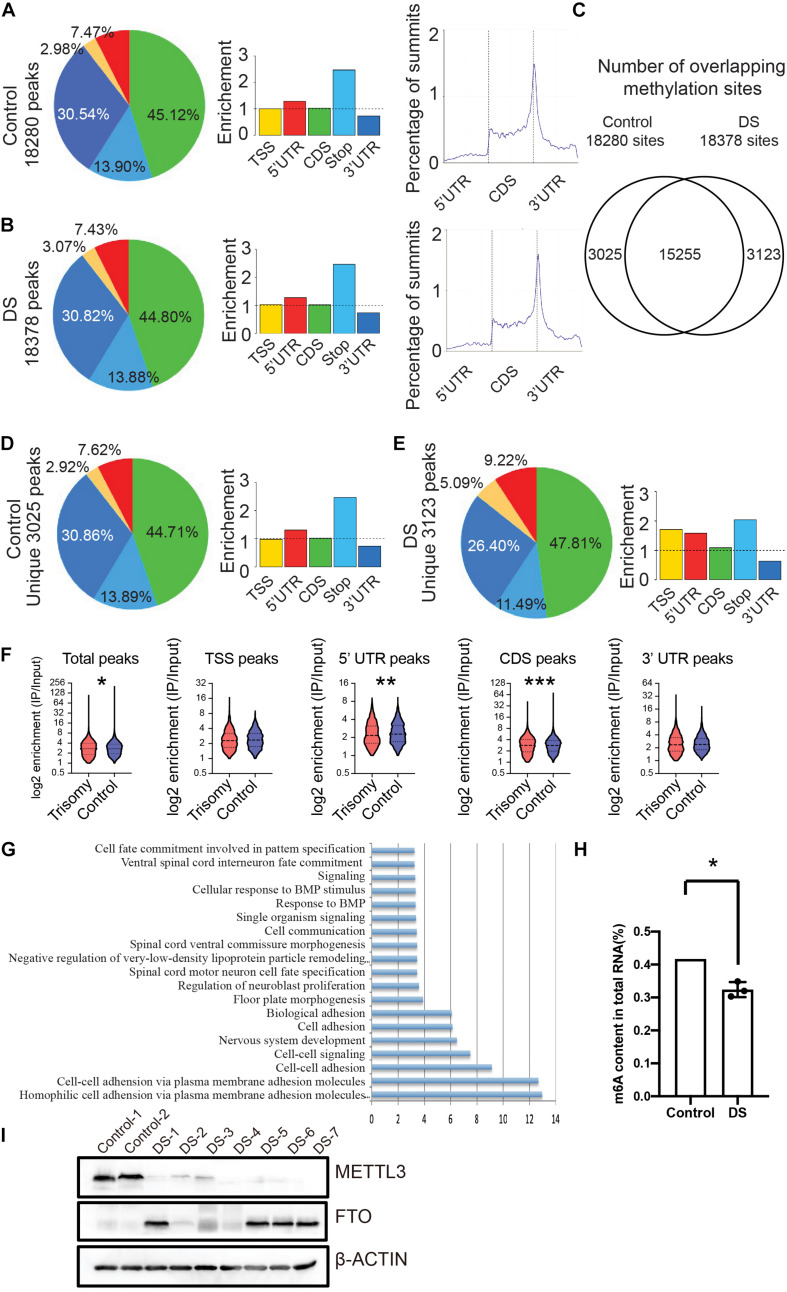
RNA m6A modification globally decreased in fetal cerebral cortex tissues of DS. **(A)** Distribution of m6A peaks identified by MeRIP-seq across the length of mRNA transcripts in control cerebral cortex samples. **(B)** Distribution of m6A peaks across the length of mRNA transcripts in cerebral cortex of DS samples. **(C)** Number of overlapping m6A sites on DS and control cerebral cortex RNAs. **(D)** Distribution of unique m6A peaks in control samples across the length of mRNA transcripts. **(E)** Distribution of unique m6A peaks in DS samples across the length of mRNA transcripts. **(F)** Violin plot depicting the transcripts containing m6A peaks on 5′ UTR was reduced in DS compare to control subjects. **(G)** GO analysis of differential m6A modified genes. **(H)** Decrease of global m6A level in total RNA isolated from DS fetal cerebral cortex compared with control via an m6A enzyme-linked immunosorbent assay kit. **(I)** Western blotting analysis of METTL3 and FTO in fetal brain tissues of seven DSs and two controls. The GAPDH was used as an internal control. *p < 0.05; **p < 0.01; ***p < 0.001 compared with the control group.

To explore the characteristics of m6A RNA modification in the DS brain, we collected cerebral cortex tissues of the fetuses diagnosed with DS and performed an optimized MeRIP combined with deep sequencing (MeRIP-seq). After mapping, a total of 18,280 m6A peaks were identified in the control group, and the distribution of m6Apeaks is mainly enriched near stop codons and in 3′ UTRs (Figure 1A), which is consistent with previous studies (Dominissini et al., 2012; Meyer et al., 2012). A total of 18,378 m6A peaks in the DS cerebral cortex were identified, and the distribution of m6A is the same as the control (Figure 1B). Next, we compared the m6A peaks of DSs with diploid controls. Most of the m6A peaks (15,255) were observed both in DSs and controls. A total of 3,123 and 3,025 unique m6A peaks were identified in DS fetuses and controls, respectively (Figure 1C). The distribution of the unique m6A peaks in the control samples is mainly enriched near stop codons and in 3′ UTRs, whereas the m6A unique peaks in 5′ UTRs increased in DSs (Figures 1D,E). We noticed that the m6A modification in DS tissues reduced genome-wide, and the reduction was more evident in 5′ UTR regions (Figure 1F). GO analysis showed that the differential m6A modified genes enriched mainly in nervous system development and neuroblast proliferation (Figure 1G).

We quantified m6A contents in total RNAs using enzyme-linked immunosorbent assays in the DSs and controls. The m6A contents in total RNA significantly decreased in DSs (Figure 1H). To explore the mechanism of the reduction of the m6A modification in DSs, we detected the protein expression of METTL3 and FTO, two critical components of m6A modification, in the DS fetal brain tissues. The western blot data clearly showed that the m6A methylase METTL3 expression in seven DS samples decreased compared with the controls (Figure 1I). While the demethylase FTO expression increased in four of seven DS samples (Figure 1I). The increased expression of the methylase and the decreased expression of the demethylase in DS fetal brain tissues may explain, at least partially, the reduction of m6A modification in DSs. These data showed that RNA m6A modification in the DS fetal cerebral cortex decreased globally.

### m6A Modification Transcripts Are Involved in Human DS Cerebral Cortex

**FIGURE 2:**
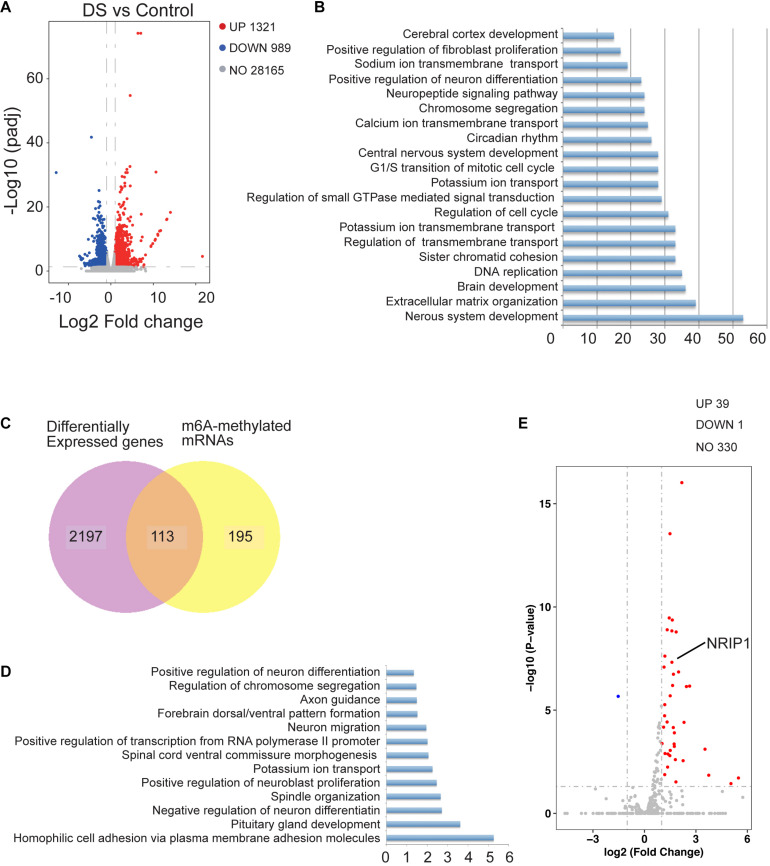
The change of m6A modification affected gene expression in human DS cerebral cortex. **(A)** Volcano plot showing log2 fold change of gene expression and corresponding adjust *p*-value. **(B)** GO analysis of differentially expressed genes identified by RNA-seq in DS cerebral cortex compared with controls. **(C)** Venn diagrams showing the overlap between differential m6A modified genes and differentially expressed genes between two groups. **(D)** GO analysis of overlapped genes identified in **(C)**. **(E)** Volcano plot showing transcriptome located on chromosome 21.

To determine the relationship between m6A modification and gene expression, we analyzed the RNA-seq data of the same cortex tissues. Many DEGs were identified, including 1,321 upregulated and 989 down-regulated genes in DSs (Figure 2A). GO analysis showed that the DEGs were related to nervous system development, brain development, and cerebral cortex development (Figure 2B). Joint analysis of MeRIP-seq and RNA-seq data identified 113 DEGs with significantly changed m6A modification (Figure 2C). The list of these overlapped genes is shown in Supplementary Table 1. These genes are mainly enriched in the negative regulation of neuron differentiation and positive neuroblast proliferation (Figure 2D). Next, we analyzed the expression of the genes located on chromosome 21. Most of the genes on chromosome 21 were upregulated in DSs, and the expression of these genes increased by about 1.5 times, which is consistent with the gene dosage effect (Figure 2E). However, there are 39 significantly upregulated genes with more than twofold expression (Figure 2E and Supplementary Table 2). Differential highly conserved chromosome 21 (Hsa21) gene expression was globally upregulated in DS tissues. We checked the m6A modification of the 39 genes and found that NRIP1 is the only one with significantly altered m6A modification. It was reported that m6A modification is associated with mRNA stability (Fu et al., 2014; Wang et al., 2014). We speculated that the overexpression of NRIP1 partly comes from the abnormality of m6A modification.

### m6A Modification of the *NRIP1* Gene Decreased in DS Cerebral Cortex

**FIGURE 3:**
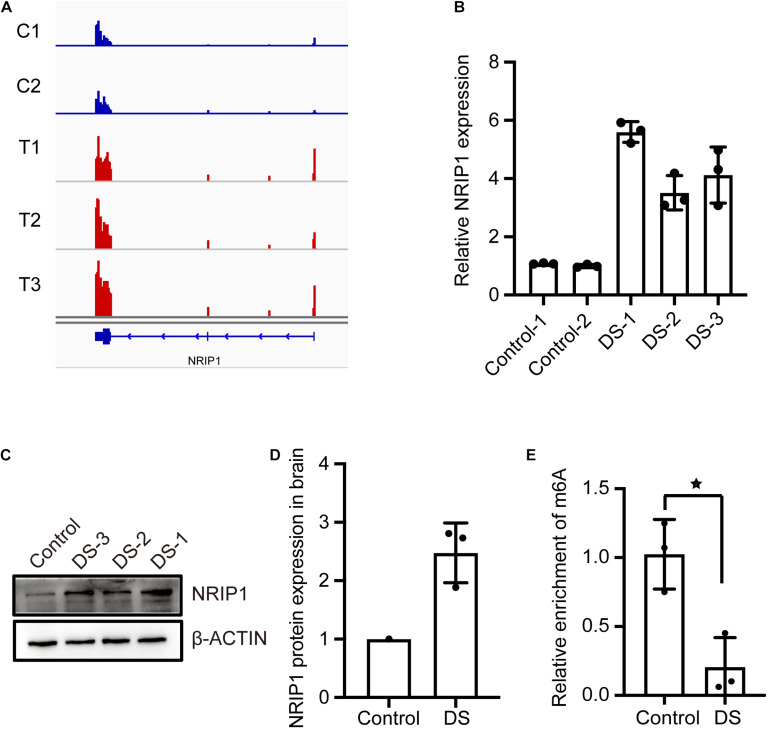
The m6A modification of NRIP1 transcripts decreased in DS cerebral cortex. **(A)** RNA-seq data showed that NRIP1 mRNA was significantly upregulated in DS compared with control. **(B)** RT-qPCR analysis confirmed that NRIP1 mRNA was increased in DS tissue. **(C,D)** NRIP1 protein level analyzed by western blotting in DS and control fetal cerebral cortex tissues. **(E)** The abundance of m6A modified NRIP1 mRNA was reduced in DS cerebral cortex compared with control sample.

Nuclear receptor-interacting protein 1, also known as receptor-interacting protein 140 (RIP140), is a protein that is encoded by the NRIP1 gene located on chromosome 21 in humans. *NRIP1* was reported to be overexpressed in many tissues from DS fetuses, and NRIP1 protein was found unregulated in the DS hippocampus (Gardiner and Costa, 2006; Conti et al., 2007; Vilardell et al., 2011; Piccoli et al., 2013; Izzo et al., 2018). The overexpression of NRIP1 is responsible for decreased respiratory efficiency and altered morphology of mitochondria in DS fetal fibroblasts (Izzo et al., 2014). The RNA-seq data showed that *NRIP1* mRNA expression was increased in DSs (Figure 3A). The qPCR assay confirmed the threefold to fivefold increase of the *NRIP1* transcript in the DS cerebral cortex (Figure 3B). The NRIP1 protein also increased significantly in DSs (Figures 3C,D). However, the m6A modification in the NRIP1 mRNA was reduced twofold to threefold in DS samples (Figure 3E). The overexpression of NRIP1 in DS cannot be explained by the dosage imbalance (Conti et al., 2007; Piccoli et al., 2013; Izzo et al., 2014). It was reported that m6A-containing mRNAs undergo rapid degradation (Lee et al., 2020). We hypothesized that the NRIP1 expression is regulated by m6A modification, and its overexpression is at least partially due to the decrease of m6A modification.

### NRIP1 mRNA Was a Target of m6A Methyltransferase METTL3

**FIGURE 4:**
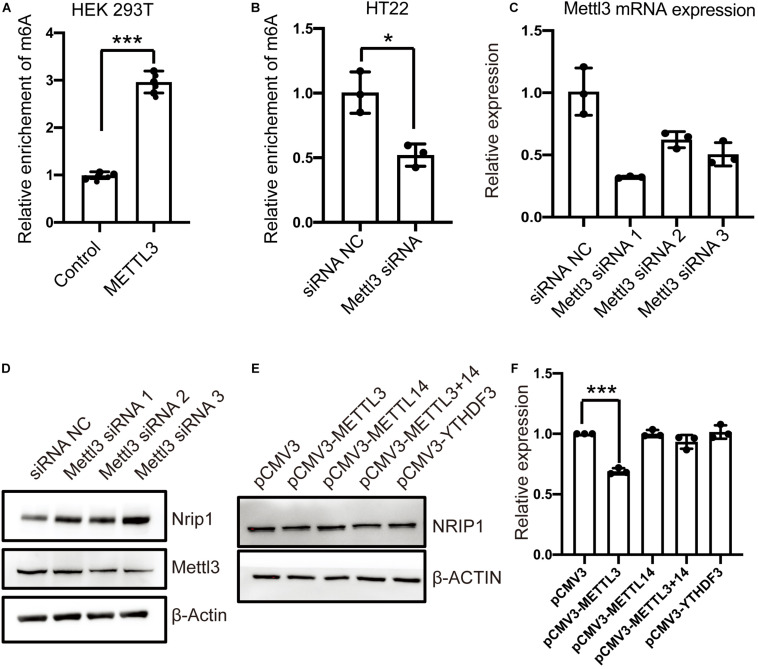
The m6A-modification of NRIP1 transcripts was regulated by METTL3. **(A)** Overexpression of METTL3 induced NRIP1 mRNA methylation in HEK293T cells. **(B)** Depletion of METTL3 decreased abundance of m6A-modified NRIP1 mRNA in HT22 cells. **(C)** RT-qPCR analysis of Mettl3 in control and Mettl3 knockdown HT22 cells. GAPDH was used as an internal control. **(D)** Western blotting analysis of Nrip1 and Mettl3 with or without Mettl3 knockdown in HT22 cells. **(E)** Western blotting analysis of NRIP1 in HEK293T cells transfected with control, METTL3, METTL14, and YTHDF3 and co-transfection of METTL3 and METTL14 plasmids. **(F)** Quantification of western blotting for different proteins. Data were presented as mean ± SD of three independent experiments. *P < 0.05; ***P < 0.001 compared with the control group.

The METTL3 and METTL14 form a heterodimer and function as a catalytic core complex known as the m6A-METTL complex (MAC). To ascertain whether NRIP1 is a substrate of METTL3, we performed MeRIP combined with qPCR detecting the NRIP1 m6A mRNA in the METTL3-overexpressed or knockdown cells. METTL3 overexpression increased the m6A modification of NRIP1 mRNA in HEK 293T cells (Figure 4A). The Mettl3 knockdown reduced *Nrip1* m6A mRNA in mouse hippocampal neuronal cells HT22 (Figure 4B). In Mettl3 knockdown HT22 cells, the *Nrip1* mRNA change was undetectable, whereas the Nrip1 protein increased compared with control (Figures 4C,D and Supplementary Figure 1). Moreover, overexpression of METTL3 significantly downregulated NRIP1 protein abundance in HEK293T cells (Figures 4E,F). YTHDF3 (YTH N6-methyladenosine RNA-binding protein 3) is an m6A-binding protein, which promotes protein synthesis of m6A modified transcripts. The reduced NRIP1 protein abundance was not observed in METTL14 or YTHDF3 overexpressed cells (Figures 4E,F).

### METTL3 Methylates NRIP1 mRNA via Recognizing the m6A Motifs in the 5′ UTR

**FIGURE 5:**
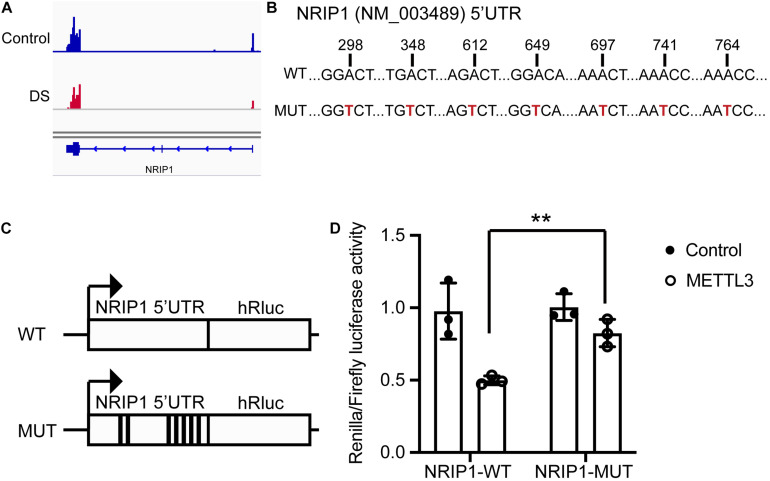
METTL3 mediated m6A modification of NRIP1 5′ UTR. **(A)** The m6A peaks are enriched in the 5′ UTR and CDS region of NRIP1 via MeRIP-seq analysis. **(B)** Several candidate m6A motifs on 5′ UTR of NRIP1 are predicted via SRAMP prediction online. **(C)** Luciferase reporter constructs containing human NRIP1 5′ UTR that have m6A motifs or mutant (A-to-G mutation) m6A sites. **(D)** Relative luciferase activities of HEK293T cells cotransfected with plasmids containing wild-type or mutant NRIP1 5′ UTR and METTL3 or control, respectively. Renilla luciferase activities were measured and normalized to firefly luciferase activity. Data were presented as mean ± SD of three independent experiments. **P < 0.01 compared with the control group.

The MeRIP-seq data showed that the m6A peaks were enriched in the 5′ UTR of NRIP1 mRNA (Figure 5A). We analyzed the sequence and found the DRACH motifs (where D = A, G, or U; R = purine; and H = A, C, or U) in the 5′ UTR region with SRAMP, an online m6A modification prediction program (Zhou et al., 2016; Figures 5B,C and Supplementary Figure 2). We inserted the 5′ UTR sequence and an m6A motif–mutated 5′ UTR (the As in the motifs were mutated to Ts) into a luciferase reporter construct, respectively, and detected the luciferase activity in transfected cells. The overexpression of the wild-type METTL3, but not catalytic mutated METTL3, substantially reduced luciferase activity around twofold in the cells transfected with the reporter containing wild-type 5′ UTR (Figure 5D and Supplementary Figure 3). The change was abrogated in the cells transfected with the mutated luciferase construct (Figure 5D). These results indicated that METTL3 regulates the expression of NRIP1 in an m6A-dependent manner.

### METTL3 Affects the Expression of NRIP1 by Regulating mRNA Stability

**FIGURE 6:**
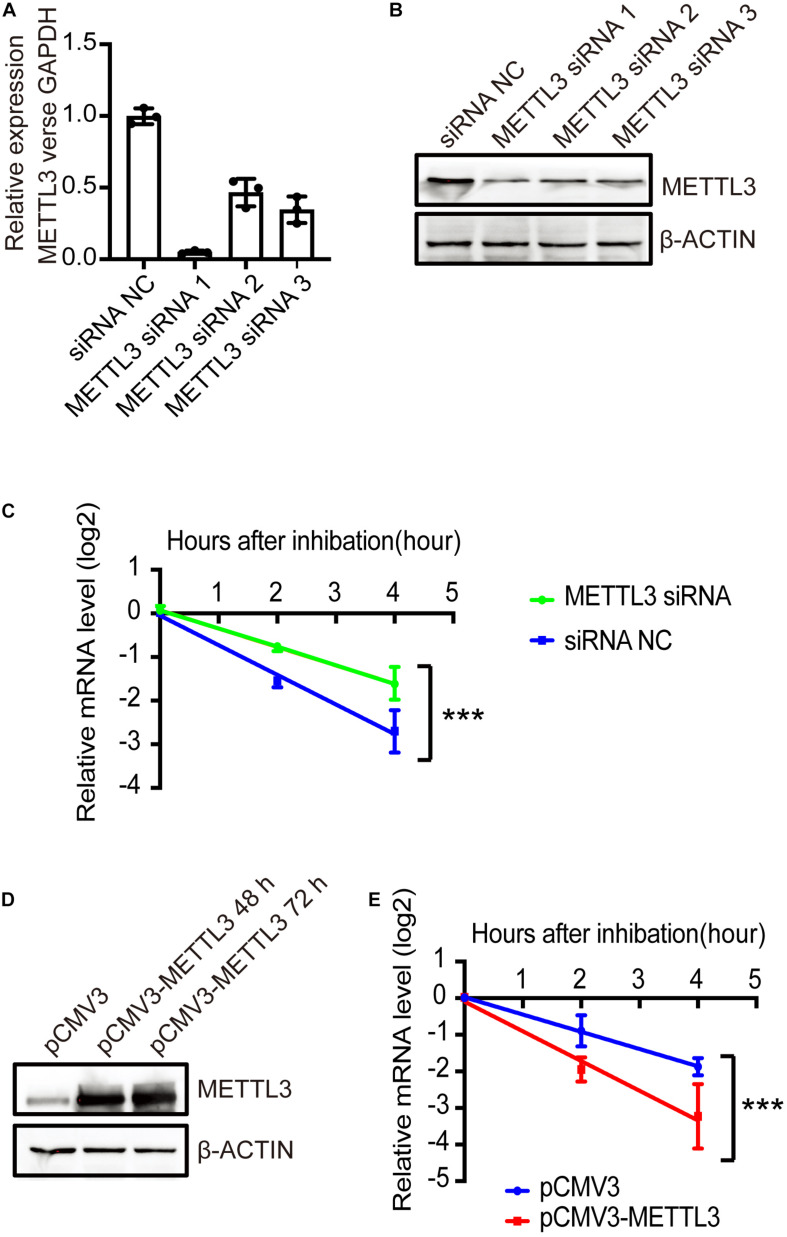
METTL3-mediated m6A regulated the stability of NRIP1 mRNA. **(A,B)** METTL3 knockdown efficiency was measured by RT-qPCR and western blotting in HEK293T cells. **(C)** The decay rate of NRIP1 mRNA was measured in HEK293T cells under siRNA NC or METTL3 siRNA after treatment of actinomycin D and comparison of remaining mRNA by quantitative RT-PCR. **(D)** Western blotting analysis of METTL3 in HEK293T cells transfected with control or METTL3 plasmid. **(E)** The decay rate of NRIP1 mRNA in HEK293T cells under control or METTL3 overexpression. Data were presented as mean ± SD of three independent experiments. ***P < 0.0001 compared with the control group.

Multiple cellular mechanisms contribute to changes in steady-state mRNA levels, and m6A-mediated mRNA decay is one of the major effects. To investigate whether m6A modification by METLL3 affects the NRIP1 mRNA decay, we measured the mRNA level in human embryonic kidney HEK 293T cells treated with the transcription inhibitor actinomycin D. Both NRIP1 and METTL3 are expressed in HEK293T, so we used this cell line to investigate the relationship between METTL3 and NRIP1. METTL3 knockdown in HEK293T cells was achieved with siRNAs, and the knockdown efficiency was confirmed by qPCR and western blotting (Figures 6A,B). The NRIP1 mRNA levels were measured at 0, 2, and 4 h after treatment with actinomycin D. METTL3 knockdown significantly increased the NRIP1 mRNA stability, and the half-life of the NRIP1 mRNA increased from 1.4 h in control cells to 2.5 h in knockdown cells (Figure 6C). Consistent with the knockdown result, the overexpression of the wild-type METTL3, but not the catalytic mutant, reduced the stability of NRIP1 mRNA (Figures 6D,E and Supplementary Figure 4). These results indicated that METTL3-mediated m6A promoted the NRIP1 mRNA decay.

## Discussion

In this study, we reported on m6A modification RNA profiles of human fetal brain cortex tissues from DS and controls. The m6A RNA profiling in DS fetal brain widely changed, indicating the critical role of m6A modification in DS pathology. We proposed a new mechanism of the overexpression of the NRIP1 gene, a Hsa21 gene, in DS fetal brain. We argue that the overexpression of NRIP1 is due to the low m6A modification and high mRNA stability in DS cells instead of the gene dosage. In DS mouse model, 18% of aneuploid genes were expressed significantly greater than theoretical value of 1.5-fold, which revealed a complex regulation of gene expression that is not only related to gene copy number (Lyle et al., 2004). In DS, the expression of APP is threefold to fourfold higher than what is expected from the 1.5-fold, suggesting that other genes on chromosome 21 directly or indirectly further upregulate APP (Wolvetang et al., 2003). Several studies including this showed that the expressions of NRIP1 in multiple tissues of DS were more than 1.5-folds, a theoretical fold of gene dosage effect (Conti et al., 2007; Piccoli et al., 2013; Izzo et al., 2014). Here we provided a new mechanism to explain the overexpression of NRIP1 beyond the gene–dose effect. The reduced m6A modification and increased mRNA stability may explain the more than 1.5-fold overexpression of the *NRIP1* gene.

Nuclear receptor-interacting protein 1 functions as a corepressor in oxidative metabolism and mitochondrial biogenesis (Nautiyal et al., 2013). Mitochondrial dysfunction in DS cells was observed, which might contribute to the DS phenotype’s severity. Overexpression of NRIP1 is associated with mitochondrial dysfunction in DS cells (Izzo et al., 2018). In fetal DS fibroblasts, siRNA-mediated NRIP1 expression attenuation restored the mitochondrial function (Izzo et al., 2014). Therefore, NRIP1 could serve as a potential therapeutic target for restoring altered mitochondrial function in DS. We observed that NRIP1 was also increased in DS fetal cortex tissues. It is further confirmed that NRIP1 was a substrate of METTL3 and that the METTL3 regulates the expression of NRIP1 in an m6A-dependent manner. In addition, METTL3 regulates the expression of NRIP1 by reducing its mRNA stability. This study enriches the knowledge about the reason for increased NRIP1 expression in DS, and the m6A modification of NRIP1 might serve as another layer of therapeutic target in DS.

N6-methyladenosine modification is highly enriched in the mammalian brain. m6A plays a critical role in the development of the mammalian brain (Widagdo and Anggono, 2018). METTL3 is the catalytic core enzyme of the m6A methyltransferase complex. The specific inactivation of Mettl3 in the mouse nervous system caused severe developmental defects in the brain. This mouse model also exhibited extended RNA half-life of apoptosis-associated genes in the loss of m6A (Wang et al., 2018). We found that the global m6A level was reduced in DS cortex tissues. Loss of m6A via METTL3 depletion–induced loss of m6A modification causes extended NRIP1 mRNA half-life, which probably further enhances the expression of NRIP1 protein. A recent study showed that m6A mRNA and METTL3 expression were elevated in the cortex of Alzheimer disease model mouse (Han et al., 2020). It was also reported that NRIP1 was involved in Alzheimer disease pathology, and the levels of NRIP1 were reduced in Alzheimer disease postmortem brains (Blondrath et al., 2016). It is an interesting question whether the NRIP1 mRNA m6A modification is also involved in Alzheimer disease.

## Conclusion

In summary, we analyzed the m6A RNA profiles of the DS cortex and proposed a new mechanism that overexpression of NRIP1 in DS brain was at least partially due to the reduced m6A-dependent mRNA decay. m6A methyltransferase METTL3 plays a crucial role in the upregulated NRIP1 in the cerebral cortex of DS patients. It helps to unravel the molecular mechanisms underlying DS brain development and leads to insights that may become therapeutically relevant.

## Data Availability Statement

The data generated for this study can be found in the Gene Expression Omnibus, Accession number GSE160691.

## Ethics Statement

The studies involving human participants were reviewed and approved by the Ethics Committee of Henan Provincial People’s Hospital, China. Written informed consent to participate in this study was provided by the participants’ legal guardian/next of kin.

## Author Contributions

BH, WS, and SL designed the experiments. WS and FY performed the lab experiments. RD conducted bioinformatics data analysis. YS and YC participated in tissue dissection, SL, WS, and BH provided the funds. WS and BH prepared the manuscript. All authors discussed the results and contributed to the final manuscript.

## Conflict of Interest

The authors declare that the research was conducted in the absence of any commercial or financial relationships that could be construed as a potential conflict of interest.
