# Supplementary figures and images for: METTL3-Mediated N6-Methyladenosine Modification Is Involved in the Dysregulation of NRIP1 Expression in Down Syndrome
Source: Front Cell Dev Biol. 2021 Apr 1;9:621374. doi: 10.3389/fcell.2021.621374 (PMC8047211; doi:10.3389/fcell.2021.621374)

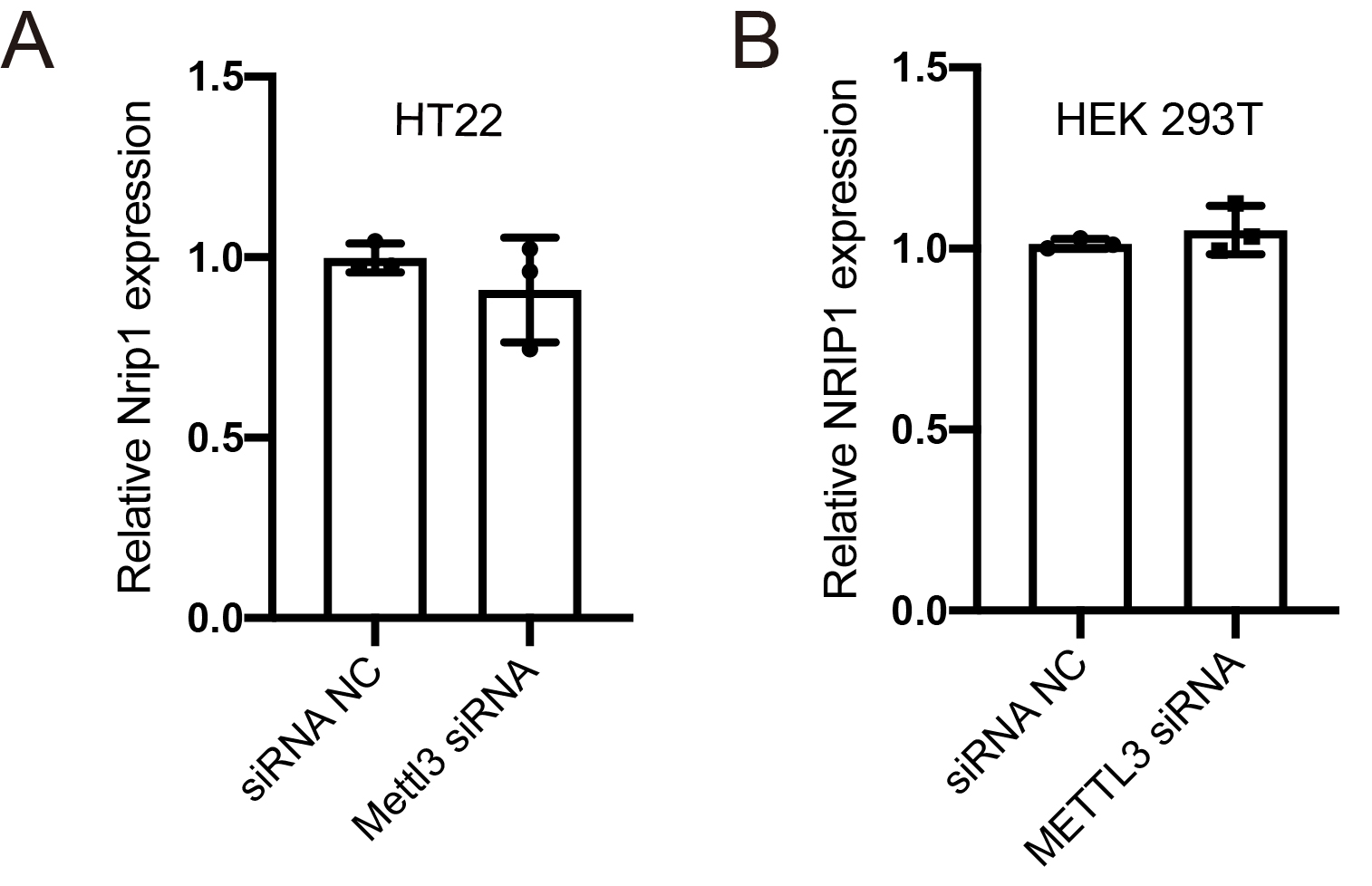

Supplement: Supplementary file 2 [file Image_1.jpg]

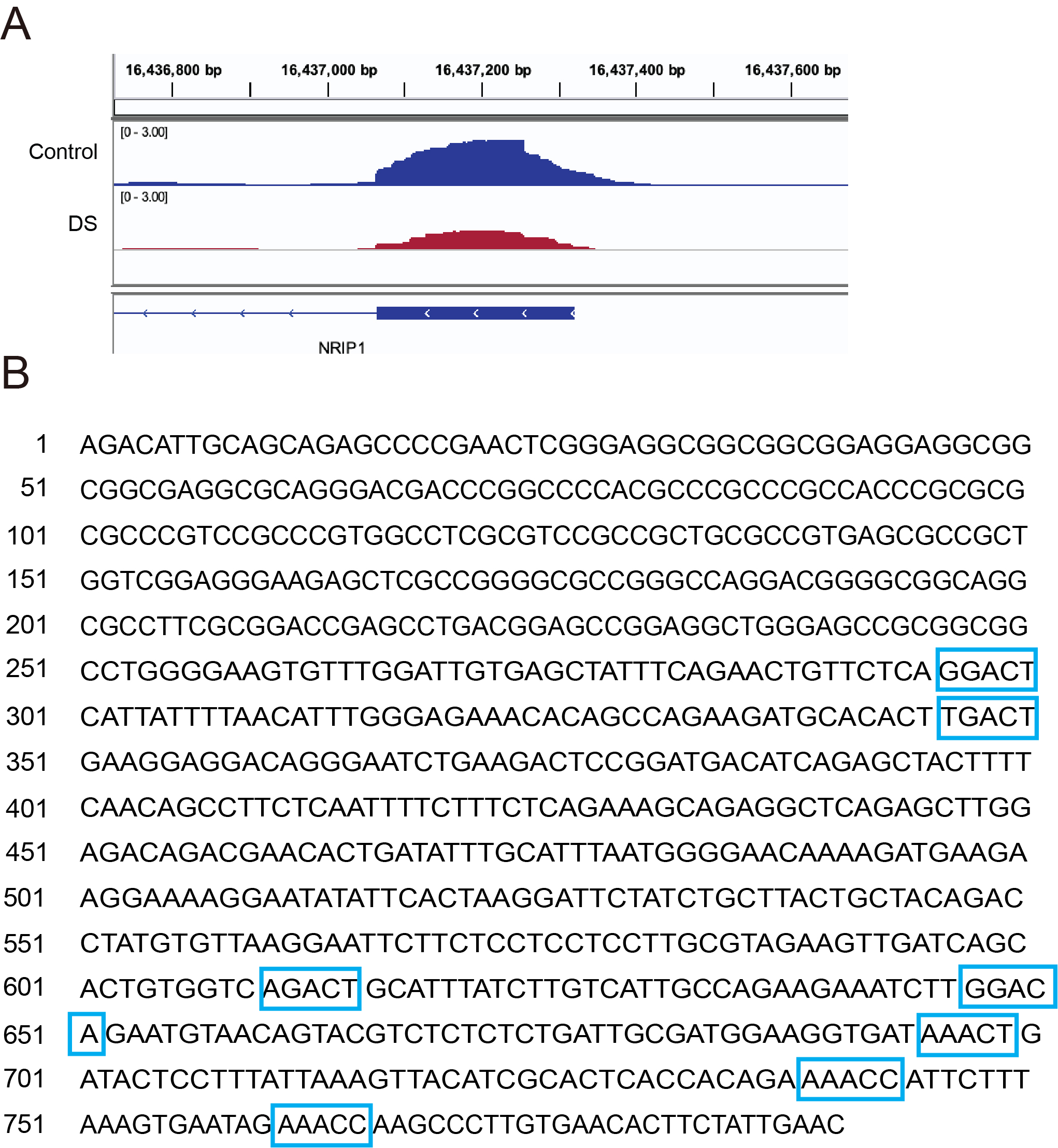

Supplement: Supplementary file 3 [file Image_2.JPEG]

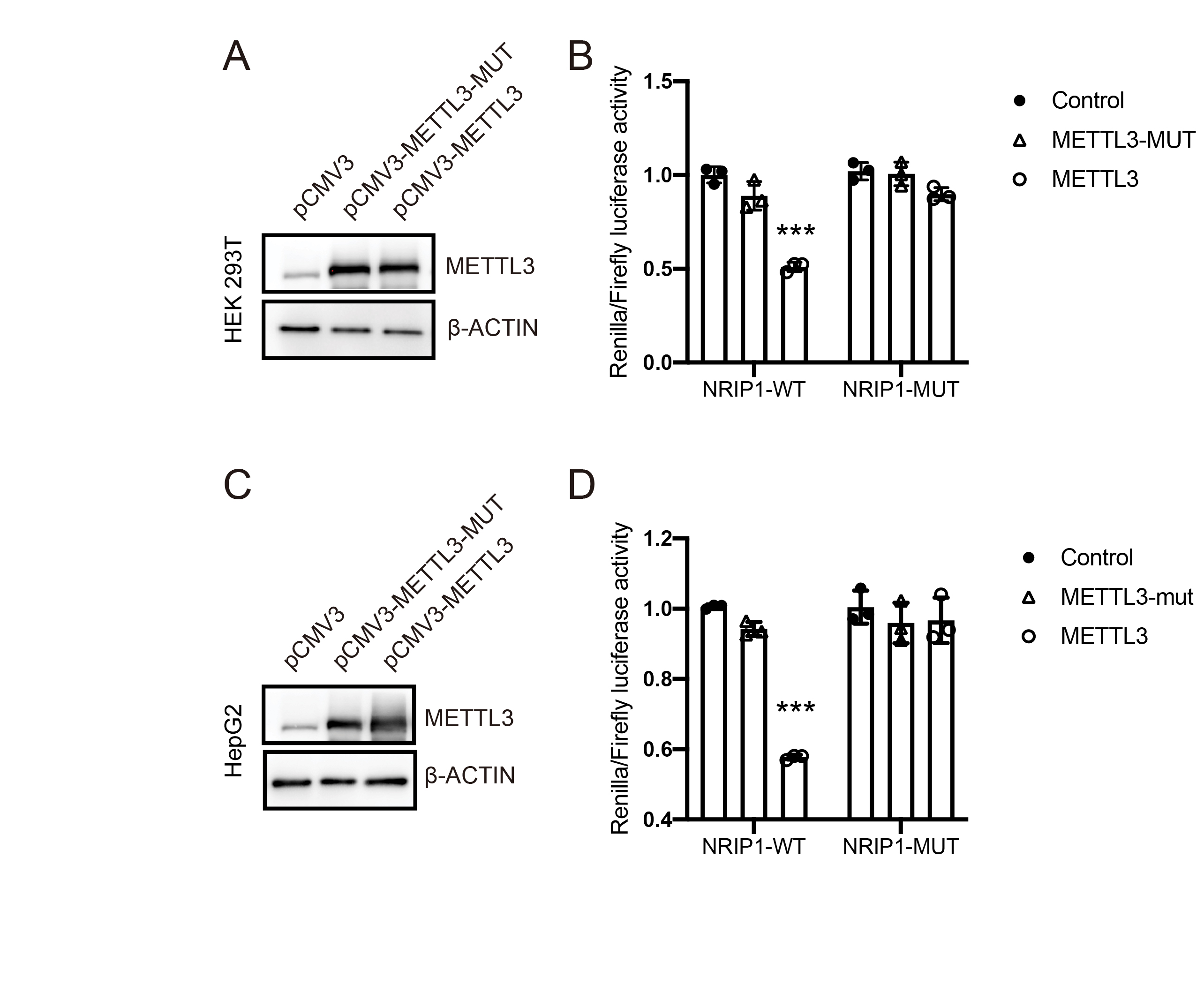

Supplement: Supplementary file 4 [file Image_3.JPEG]

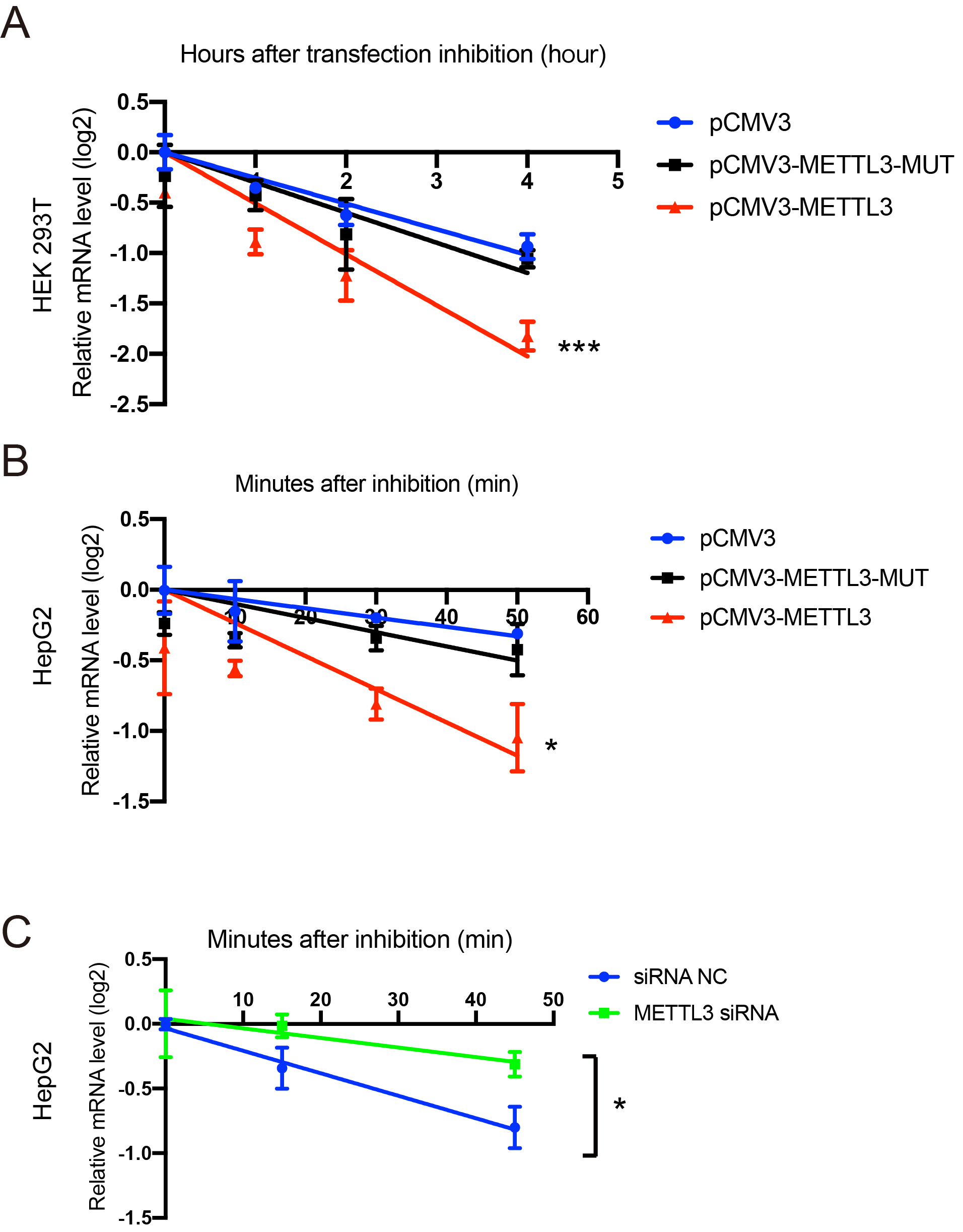

Supplement: Supplementary file 5 [file Image_4.JPEG]
